# Modulation of the Gut Microbiota by *Sihocheonggan*-*Tang* Shapes the Immune Responses of Atopic Dermatitis

**DOI:** 10.3389/fphar.2021.722730

**Published:** 2021-09-20

**Authors:** Jaemoo Chun, So Min Lee, You Mee Ahn, Min-Gyung Baek, Hana Yi, Sarah Shin, Jeeyoun Jung

**Affiliations:** ^1^ KM Science Research Division, Korea Institute of Oriental Medicine, Daejeon, South Korea; ^2^ KM Convergence Research Division, Korea Institute of Oriental Medicine, Daejeon, South Korea; ^3^ Interdisciplinary Program in Precision Public Health, Korea University, Seoul, South Korea; ^4^ Department of Public Health Sciences, Korea University, Seoul, South Korea; ^5^ School of Biosystems and Biomedical Sciences, Korea University, Seoul, South Korea

**Keywords:** Sihocheonggan-Tang, atopic dermatitis, gut microbiome, short-chain fatty acids, immune response

## Abstract

Atopic dermatitis (AD) is a chronic inflammatory skin disease characterized by complex immune dysregulation and closely related to the gut microbiome. The present study investigated the microbiome-mediated effect of *Sihocheonggan-Tang* (SHCGT) on AD-like symptoms induced by 2,4-dinitrochlorobenzene (DNCB) in BALB/c mice. DNCB was applied regularly to the ear and dorsal skin of BALB/c mice, and SHCGT was administered orally daily for 2 weeks. The composition of the gut microbiota was analyzed using 16S rRNA sequencing, and the effect of gut microbiome-derived metabolites, specifically short-chain fatty acids (SCFAs), was evaluated in tumor necrosis factor-alpha (TNF-α)- and interferon-gamma (IFN-γ)-treated HaCaT cells. SHCGT alleviated DNCB-induced symptoms of AD and the immune response to AD by decreasing the plasma immunoglobulin E level and splenic interleukin-4, interleukin-10, TNF-α, and IFN-γ levels. The gut microbiome composition and the damaged gut epithelial barrier in mice with AD were also significantly altered by SHCGT, and the reduced SCFA levels therein were elevated. We found that SFCAs directly inhibited the mRNA expression of IL-6 and ICAM-1 in TNF-α- and INF-γ-treated HaCaT cells. The finding that SHCGT regulates the gut microbiome and improves DNCB-induced AD in mice suggests that this herbal medicine has therapeutic potential in patients with AD.

## Introduction

Atopic dermatitis (AD) is a chronic inflammatory skin disease caused by immune system dysfunction and environmental stress (Akdis et al., 2000). It is typically characterized by increases in T helper (Th)2 cell-mediated inflammatory responses, including the release of immunoglobulin E (IgE), interleukin (IL)-4, IL-5, and IL-13 in the acute phase, whereas an increasing proportion of Th1 cells accounts for the chronic phase of the immune response (Biedermann et al., 2015; Moreno et al., 2016). AD is usually treated with topical anti-inflammatory or immunosuppressive agents, such as antihistamines and steroids. However, many of the treatments available focus on reducing the frequency and severity of symptoms and may have severe adverse effects. Therefore, a new approach is needed to treat AD effectively (Paller et al., 2017).

The microbiome is a complex collection of microorganisms that colonize the gut and play a crucial role in the health of the host, including digestion of dietary fiber, production of metabolites, and regulation of the immune response (Makki et al., 2018). Maintaining a correctly balanced microbiota in the gut is important for homeostasis in the host immune response, and disruption of the balance of the gut microbiota could cause various diseases, including allergic and inflammatory disorders (Kamada et al., 2013). Therefore, modulating the composition of the gut microbiome is a promising way of overcoming the adverse effects or limitations of current treatments for these diseases. The gut microbiome could directly or indirectly communicate with the skin as one of the important modulators in the gut-skin axis. The beneficial effects of gut microbiota by probiotics on skin diseases have been demonstrated in animal studies (Salem et al., 2018) Moreover, emerging clinical evidence suggests that AD is associated with an imbalance in bacterial species in the gut (Song et al., 2016) and that treatment with probiotics or prebiotics prevents the development of AD or reduces its severity (Isolauri et al., 2000; Grüber et al., 2010; Foolad et al., 2013; Lin et al., 2019). Thus, regulation of gut microbiome balance could be promising strategy for the treatment and prevention of AD.

Herbal medicines can also restore the composition and beneficial effects of the gut microbiota, thereby improving immune-related diseases, including autoimmune disorders, allergies, and asthma (Richards et al., 2016; An et al., 2017). *Sihocheonggan-Tang* (SHCGT) consists of 15 herbs (*Bupleurum falcatum* L.*, Angelica gigas* Nakai*, Paeonia lactiflora* Pall.*, Ligusticum striatum* DC., *Rehmannia glutinosa* (Gaertn.) DC., Coptis chinensis Franch.*, Scutellaria baicalensis* Georgi*, Phellodendron chinense* C.K.Schneid.*, Gardenia jasminoides* J.Ellis*, Forsythia suspensa* (Thunb.) Vahl*, Platycodon grandiflorus* A. DC.*, Arctium lappa* L.*, Trichosanthes kirilowii* Maxim.*, Mentha arvensis* L., and *Glycyrrhiza uralensis* Fisch.). It originates from Yizong Jinjian and has been used to treat inflammatory skin diseases, including AD, in clinical settings in Korea (An et al., 2017). However, the mechanism by which this drug exerts its therapeutic effect in skin diseases such as AD remains to be elucidated.

This study assessed the effects of SHCGT on symptoms of AD, infiltration of immune cells into the dermis and epidermis, and cytokine expression levels in plasma and the spleen in a mouse model of AD. In addition, we investigated changes in the composition of the gut microbiome and gut microbiome-derived metabolites. This study aimed to clarify the gut microbiome-mediated effect involved in the pathogenesis of AD and provide effective AD treatment with SHCGT. This is the first study to demonstrate the relationship between gut microbiome, their producing metabolites, and AD symptoms.

## Materials and Methods

### Analysis of SHCGT by UPLC/Q-TOF MS

SHCGT was purchased from GMP (Good Manufacturing Practice) pharmaceutical factory of Hanpoong Pharm & Foods Co. Ltd. (Jeonju, Republic of Korea). The voucher number of SHCGT in this study is #17083 and stored in Korea institute of Oriental Medicine. According to the manufacturer, one dose of SHCGT consists of Bupleuri radix (0.67 g), Forsythiae Fructus (0.5 g), Cnidii Rhizoma (0.5 g), Trichosanthis Radix (0.5 g), Scutellariae Radix (0.5 g), Gardeniae Fructus (0.5 g), Paeoniae Radix (0.5 g), Arctii Fructus (0.5 g), Coptidis Rhizoma (0.5 g), Angelicae Gigantis Radix (0.5 g), Platycodi Radix (0.5 g), Rehmanniae Radix Preparata (0.5 g), Menthae Herba (0.5 g), Phellodendri Cortex (0.5 g), Glycyrrhizae Radix (0.5 g) (Korea pharmaceutical information center, 2000; Supplementary Table S1). Details about preparation of SHCGT and species of fifteen herbal medicines are given in the Supplementary Methods.

In this study, we further verified the chemical characteristics of SHCGT using ultra-performance liquid chromatography (UPLC; Waters Corp., Milford, MA, United States) combined with quadrupole time-of-flight mass spectrometry (Q-TOF MS; Impact HD; Bruker, Bremen, Germany; Supplementary Figure S1).

Ten milligrams of SHCGT was mixed with 1 ml of methanol-water solution (50:50, v/v). The sample was vortexed for 1 min and then centrifuged at 12,500 ×*g* and 25°C for 20 min. The supernatant was collected and filtered using a Millex®-LG filter with a 0.20-μm pore size (Millipore, Billerica, MA, United States). A UPLC BEH C18 column (100 × 2.1 mm, 1.7-μm particle size; Waters Corp.) was used to separate the SHCGT at 40°C.

The UPLC/Q-TOF MS data were processed using MS-Dial. The chemical components of SHCGT were identified based on the retention time, m/z, and MS fragment pattern using all publicly available mass spectral databases obtained from RIKEN (http://prime.psc.riken.jp/Metabolomics_Software/MS-DIAL/), which include the MS/MS records of MassBank, ReSpect, GNP, Fiehn HILIC, CASMI2016, RIKEN PlaSMA authentic standards, and RIKEN PlaSMA bio-MS/MS (Tsugawa et al., 2015). Details of the analysis of chemical components using UPLC-Q-TOF MS are given in the Supplementary Methods.

### Animal Studies

Animal experiments were executed in accordance with the guidelines of the Institutional Animal Care and Use Committee of the Korea Institute of Oriental Medicine (Approval number #18-021). Six-week-old BALB/c mice were purchased from Saeron Bio Co. (Gyeonggi-do, Republic of Korea) and housed at 20–22°C with a relative humidity of 40–60% on a 12:12-h light/dark cycle in a specific pathogen-free animal facility at the Korea Institute of Oriental Medicine.

**FIGURE 1:**
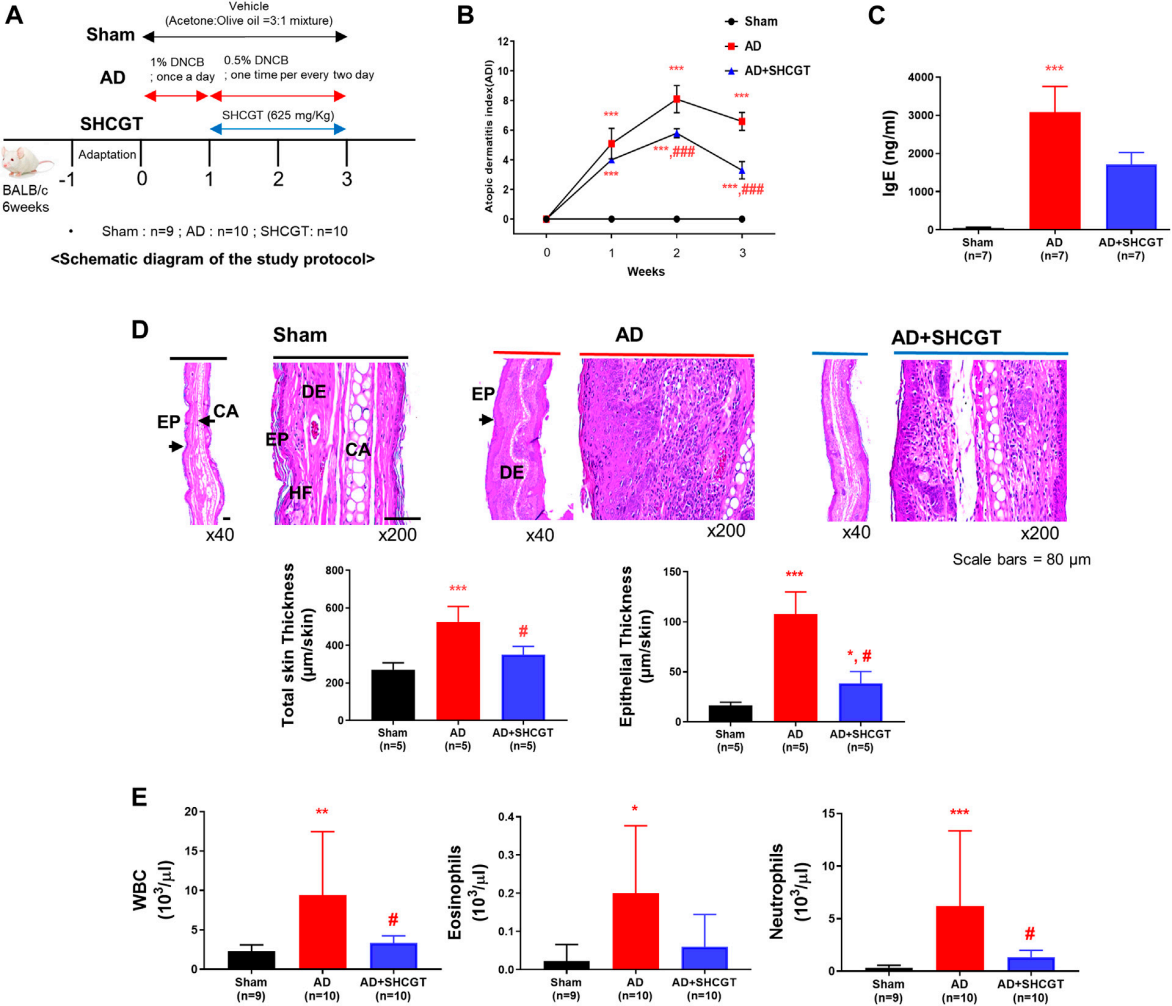
Oral administration of SHCGT suppresses DNCB-induced AD. **(A)** Timeline for induction of AD and treatment with SHCGT. **(B)** The severity of DNCB-induced AD was evaluated based on four symptoms (erythema/edema, pruritus/dryness, erosion, and lichenification) and scored as 0 (none), 1 (mild), 2 (moderate), or 3 (severe). **(C)** Plasma IgE. **(D)** Ear skin specimens stained with hematoxylin. Total skin and epithelial thickness were evaluated by histological analysis. **(E)** Total WBC, eosinophil, and neutrophil levels. *p < 0.05, **p < 0.01, ***p < 0.001 vs. sham; ^#^ *p* < 0.05, ^###^ *p* < 0.001 vs. AD. AD, atopic dermatitis; DNBC, 2,4-dinitrochlorobenzene; IgE, immunoglobulin E; SHCGT, *Sihocheonggan-Tang*; WBC, white blood cell.

After acclimatization for 1 week, the mice were randomized to a sham (control) group (*n* = 9), an AD group (*n* = 10), or an AD + SHCGT group (*n* = 10). All mice were shaved, and AD was induced by DNCB (Sigma-Aldrich, St Louis, MO, United States). During the first week of the AD induction period, 1% DNCB in acetone/olive oil (3:1) was applied to the ear and dorsal skin daily, followed by 0.5% DNCB in acetone/olive oil (3:1) on alternate days for the next 2 weeks (Figure 1A). SHCGT or phosphate-buffered saline (100 μL) was administered to the mice by intragastric gavage once daily (Figure 1A). The severity of atopic symptoms was evaluated based on a previous method (Suto et al., 1999). Briefly, the AD score was measured as the sum of five symptoms, including erythema/edema, pruritus/dryness, erosion, and lichenification (0, none; 1, mild; 2, moderate; 3, severe).

The dose of SHCGT in this study is determined based on the clinical usage. The Ministry of Food and Drug Safety of Korea has approved the efficacy of SHCGT for the treatment of eczema by taking 3,000 mg/day for adult (reference body weight, 60 kg), which can be converted to 625 mg/kg for mouse when applied proportional constant of 0.08 (Nair and Jacob, 2006). Thus, we administrated 625 mg/kg of SHCGT in this study, and confirmed dose-dependent effect in additional experiments (Supplementary Figure S2). Details about additional experiments were described in Supplementary Results and Supplementary Figure S2.

### Measurement of IgE

Blood samples were drawn from the sham, AD and AD+SHCGT groups, and plasma was collected by centrifugation at 2,000 ×*g* for 15 min at 4°C. It was stored at −80°C until the test. Plasma concentrations of total IgE were measured using an enzyme-linked immunosorbent assay kit (Bethyl Laboratories Inc., Montgomery, TX, United States) according to the manufacturer’s instructions.

### Isolation of Splenocytes and Measurement of Cytokines

The abdominal area was sterilized with 70% ethanol. The spleen was removed aseptically via an incision, and the connective tissues were removed. The spleen was then rinsed in RPMI-1640 medium (Gibco; Thermo Fisher Scientific, San Jose, CA, United States) and placed in a Petri dish containing fresh RPMI-1640 medium. The spleen cells were teased out using forceps and forced through a 22-G needle and then through a 26-G needle three times each. The cells were then transferred to a tube and centrifuged at 200 ×*g* for 10 min to pellet the cells. The spleens were pressed through a sterile Falcon cell strainer (BD Biosciences, Franklin Lakes, NJ, United States). Red blood cell lysis buffer (BioLegend, San Diego, CA, United States) was added to the cell suspension to remove the red blood cells. The spleen cells were centrifuged, suspended in complete RPMI-1640 medium with 10% fetal bovine serum and 1% antibiotics (penicillin, streptomycin, gentamicin; Gibco), and maintained at 37°C in a humidified incubator with 5% CO_2_. After incubation for 24 h in a CO_2_ incubator, the culture supernatants were assayed for cytokines. IL-4, IL-10, tumor necrosis factor-alpha (TNF-α), and interferon-gamma (IFN-γ) levels were analyzed using the Bio-Plex Pro Mouse Cytokine Th1/Th2 Assay kit (Bio-Rad Laboratories, Hercules, CA, United States).

### Immunohistochemical Staining

Samples of equal size were taken from the ear skin, lymph nodes, and small intestine and fixed in 10% neutral buffered formalin for 24 h at 4°C. Paraffin-embedded tissue sections (3–4-μm thick) were stained with hematoxylin and eosin. The sections were stained with toluidine blue for detection of mast cells in the skin and periodic acid-Schiff (PAS) for detection of goblet cells in the small intestine. Intercellular adhesion molecule-1 (ICAM-1; ab119871, Abcam, Cambridge, MA, United States) and thymic stromal lymphopoietin (TSLP; ab115700, Abcam) in the epidermis and dermis of skin, Foxp3 (FJK-16s, Thermo Fisher) in lymph nodes, and tight junction markers, such as claudin-5 (4C3C2, Thermo Fisher), occludin (OC-3F10, Thermo Fisher), and zonula occludens (ZO)-1 (61–7,300, Thermo Fisher), in the tissues of the small intestine were observed by avidin-biotin-peroxidase complex-based immunohistochemical staining. The histological profiles of individual cross-sections of ear skin were observed under a light microscope (Eclipse 80i, Nikon, Tokyo, Japan).

### Analysis of the Gut Microbiota

Immediately before the mice were euthanized, fresh fecal samples were collected and stored at −80°C until processing. A FastDNA Spin Kit for Soil (MP Biomedicals, Santa Ana, CA, United States) was used to isolate the fecal DNA. Fecal microbial profiles were determined by analysis of the V3–V4 region of the bacterial 16S rRNA gene using the 250-bp paired-end read strategy on the MiSeq sequencing system (Illumina, San Diego, CA, United States).

### Analysis of Short-Chain Fatty Acids

Forty milligrams of stool was diluted in 900 μL of 0.2 M phosphate buffer solution (0.2 M Na_2_HPO_4_, 0.2 M NaH_2_PO_4_ in D_2_O, pH 7.0). After homogenization at 14,000 ×*g* for 10 min, the extract (700 μL) was transferred to 5-mm nuclear magnetic resonance (NMR) tubes for analysis. One-dimensional 1H NMR spectra were acquired using an Ascend 800-MHz AVANCE III HD Bruker spectrometer (Bruker BioSpin AG) with a triple-resonance 5-mm CPTIC cryogenic probe. Bruker standard 1D nuclear Overhauser enhancement spectroscopy (NOESY)-presat (noesypr1d) pulse sequences were used as follows: relaxation delay, 90° – short delay, 90° – mixing, 90°– Acq, with a relaxation delay of 4.0 s, short delay = 11.3 μs, *n* = 128, dummy scans = 4, acquisition time = 2.0 s, and mixing time = 10 ms. The 800-MHz Chenomx library (ver. 7.1, Chenomx, Edmonton, AB, Canada) was used to identify short-chain fatty acids (SCFAs), and two-dimensional total correlation spectroscopy and heteronuclear single quantum correlation spectroscopy were also used to assign the individual compounds. In addition, ambiguous peaks due to peak overlap were confirmed by spiking with standard compounds. SCFA metabolites were quantified using Chenomx, which uses the concentration of trimethylsilylpropanoic acid to determine the concentrations of individual compounds (Supplementary Table S3).

### Culture of HaCaT Cells and Treatment With Short-Chain Fatty Acids

HaCaT cells (human keratinocytes) were obtained from the American Type Culture Collection (Rockville, MD, United States). The cells were cultured in Dulbecco’s modified Eagle’s medium containing 10% heat-inactivated fetal bovine serum, 100 μg/ml streptomycin, and 100 U/mL penicillin in an incubator with a humidified atmosphere of 5% CO_2_ at 37°C. Albiflorin, baicalin, geniposide, glycyrrhizic acid, and paeoniflorin were purchased from Wako Pure Chemical Industries (Osaka, Japan). Apigenin and luteolin purchased from Chengdu Biopurify Phytochemicals Ltd. (Chengdu, China). Berberine hydrochloride was purchased from Shanghai Sunny Biotech Co., Ltd. (Shanghai, China). Physcion was purchased from ChemNorm Biotech Co., Ltd. (Wuhan, China). The purity of compounds used in this study was higher than 98%. A cell counting kit-8 assay (CCK-8, Dojindo, Kumamoto, Japan) was performed to determine the effect of noncytotoxic concentrations of SHCGT, SCFAs and compounds on the viability of HaCaT cells. Briefly, HaCaT cells were treated with SHCGT (125–1,000 μg/ml), compounds (12.5–100 µM), and SCFAs (isobutyrate, propionate, and valerate in the range of 1.25–10 mM) for 24 h. Next, 10 µL of CCK-8 reagent was added to each well and incubated for 2 h. The absorbance of each well was detected at 450 nm using a Benchmark Plus microplate reader (Bio-Rad), and determined the proper concentrations of isobutyrate, propionate, and valerate.

To evaluate the anti-inflammatory effect on keratinocytes, HaCaT cells were pretreated with SHCGT, compounds, and SCFAs at predetermined concentrations in the CCK-8 assay (SHCGT, 125–1,000 μg/ml; Albiflorin, 100 μM; baicalin, 50 μM; geniposide, 100 μM; glycyrrhizic acid, 100 μM; paeoniflorin, 100 μM; apigenin, 25 μM; luteolin, 25 μM; berberine hydrochloride, 50 μM; physcion, 100 μM; isobutyrate, 2.5 mM; propionate, 2.5 mM; and valerate, 2.5 mM) for 6 h and then stimulated with TNF-α (20 ng/ml) and IFN-γ (20 ng/ml) for an additional 18 h.

### Quantitative Reverse Transcription-Polymerase Chain Reaction

Total RNA was isolated from HaCaT cells using the RNeasy Kit (Qiagen, Hilden, Germany). Total RNA (1 μg) was converted into cDNA using the High-Capacity cDNA Reverse Transcription Kit (Applied Biosystems, Foster City, CA, United States). Quantitative polymerase chain reaction (PCR) was performed on a CFX Connect Real-Time PCR Detection System (Bio-Rad) using iTaq Universal SYBR Green Supermix (Bio-Rad). The forward primer used for IL-6 was 5-TAC CCC CAG GAG AAG ATT CC-3, and the reverse was 5-GCC ATC TTT GGA AGG TTC AG-3′. The forward primer used for ICAM-1 was 5-GCT GGT GAC ATG CAG CAC C-3, and the reverse was 5-CTC CTC ACC AGC ACC GTG G G-3′. All reactions were performed in triplicate. All gene levels were calculated by comparative delta Ct and normalized using the GAPDH gene level.

### Analysis of Gut Microbiome Data

QIIME (Caporaso et al., 2010) with the EzBioCloud 16S rRNA gene sequence database was utilized to analyze the sequencing data (Yoon et al., 2017). The threshold of operational taxonomic units was 97%. OTU data were imported to Microbiomeanalyst (https://www.microbiomeanalyst.ca/; Chong et al., 2020) and subjected to following data analysis. The Shannon index was calculated to determine the alpha diversity, and principal coordinates analysis was performed using weighted normalized UniFrac distances. In addition, linear discriminant analysis effect size (LEfSe) (Segata et al., 2011), a biomarker discovery method, was used to determine the microbiota that best characterized each study group based on the *p*-value (<0.05) and linear discriminant analysis score (>3). Pattern search analysis was also performed to identify the microbiota that were correlated with a particular pattern in abundance (Sham-AD-AD+SHCGT; 1-2-1). We, then, executed the parametric and non-parametric ANOVA tests with post-hoc test to compare the microbiota among groups. Details about the parametric and non-parametric ANOVA tests were in 2.11 Statistical Analysis part.

### Statistical Analysis

All the experimental data are presented as the mean ± standard deviation and were analyze differences among the three groups using one-way analysis of variance (ANOVA) followed by Tukey’s post hoc test. However, nonparametric analysis was performed using and the Kruskal-Wallis test followed by Dunn’s multiple comparison test, when normality and homogeneity of variance was not satisfied through the normality and homogeneity of variance test. The statistical analyses were performed using GraphPad Prism (version 9.0.2, GraphPad Software, San Diego, CA). A *p*-value < 0.05 was considered statistically significant.

## Results

### Chemical Components in SHCGT

**FIGURE 2:**
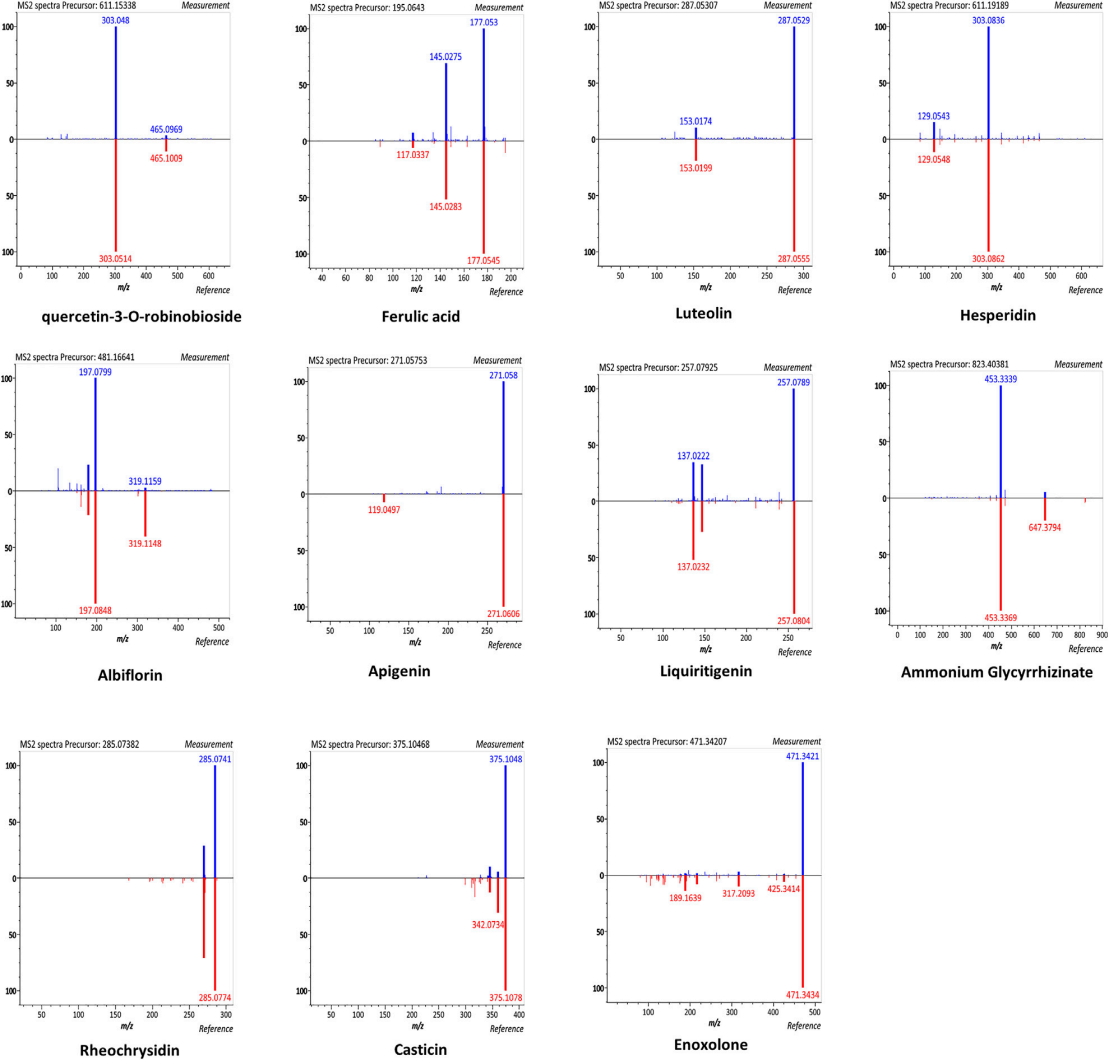
The mirror plots of secondary metabolites in SHCGT. The mirror plots that illustrate MS/MS spectral match of measured peaks of secondary metabolites in SHCGT and reference peaks for authentic standard compounds.

According to the manufacturer, SHCGT contains five chemical ingredients (10 mg of paeoniflorin, 25 mg of berberine, 22.5 mg of geniposide, 50 mg of baicalin, and 12.5 mg of glycyrrhizic acid in 3 g of SHCGT) as marker compounds (Korea pharmaceutical information center, 2000). In this study, we further identified the chemical components of SHCGT based on UPLC-Q-TOF MS analysis. A total of 2,221 features were detected, and 49 metabolites were putatively matched with reference peaks of primary and secondary metabolites (Supplementary Figure S1). In particular, quercetin-3-O-robinoside, ferulic acid, luteolin, hesperidin, albiflorin, apigenin, liquiritigenin, ammonium glycyrrhizinate, rheochrysidin, casticin, and enoxolone were identified according to relative retention time, exact MS, and MS/MS fragments (Figure 2). Furthermore, we confirmed the existence and concentration of eight bio-compounds, such as ferulic acid, luteolin, hesperidin, albiflorin, apigenin, ammonium glycyrrhizinate, rheochrysidin, and casticin, with standard compounds (Data not shown).

### Effect of SHCGT on DNCB-Induced AD in Mice

To investigate the effect of SHCGT on AD, DNCB was regularly applied to the ear and dorsal skin of BALB/c mice, and SHCGT was administered orally daily for 2 weeks. On days 7–21, atopic dermatitis index (ADI) scores were significantly higher in BALB/c mice treated with DNCB than in sham mice (Figure 1B). On days 14–21, the decrease in the ADI score was steeper in the AD + SHCGT group than in the AD group [from 8.1 to 6.6 (AD group) and from 5.8 to 3.3 (AD + SHCGT group)]; furthermore, the ADI score was significantly lower in the AD + SHCGT group than in the AD group on day 14 and day 21 (Figure 1B). IgE levels were also significantly higher in the AD group than in the sham group (Figure 1C). Histological analysis revealed that ear skin tissue was thicker (due to epidermal keratinocyte hyperplasia) in the AD and AD + SHCGT groups than in the sham group, but ear skin in AD + SHCGT mice was thinner than that in AD mice (Figure 1D), indicating a favorable inhibitory effect of SHCGT on DNCB-induced thickening of the epidermal tissue.

We also measured the numbers of circulating white blood cells (WBCs), eosinophils, and neutrophils. The AD group had 4.1-fold, 9.0-fold, and 18.0-fold higher WBC, eosinophil, and neutrophil counts, respectively, than the sham group (Figure 1E). However, compared with the AD group, treatment with SHCGT reduced the WBC, eosinophil, and neutrophil counts by 35.5, 30.0, and 21.5%, respectively (Figure 1E). Furthermore, the ADI index, the percentage of neutrophil, and corticosterone were changed in dose-dependent manners (Supplementary Results and Supplementary Figure S2).

**FIGURE 3:**
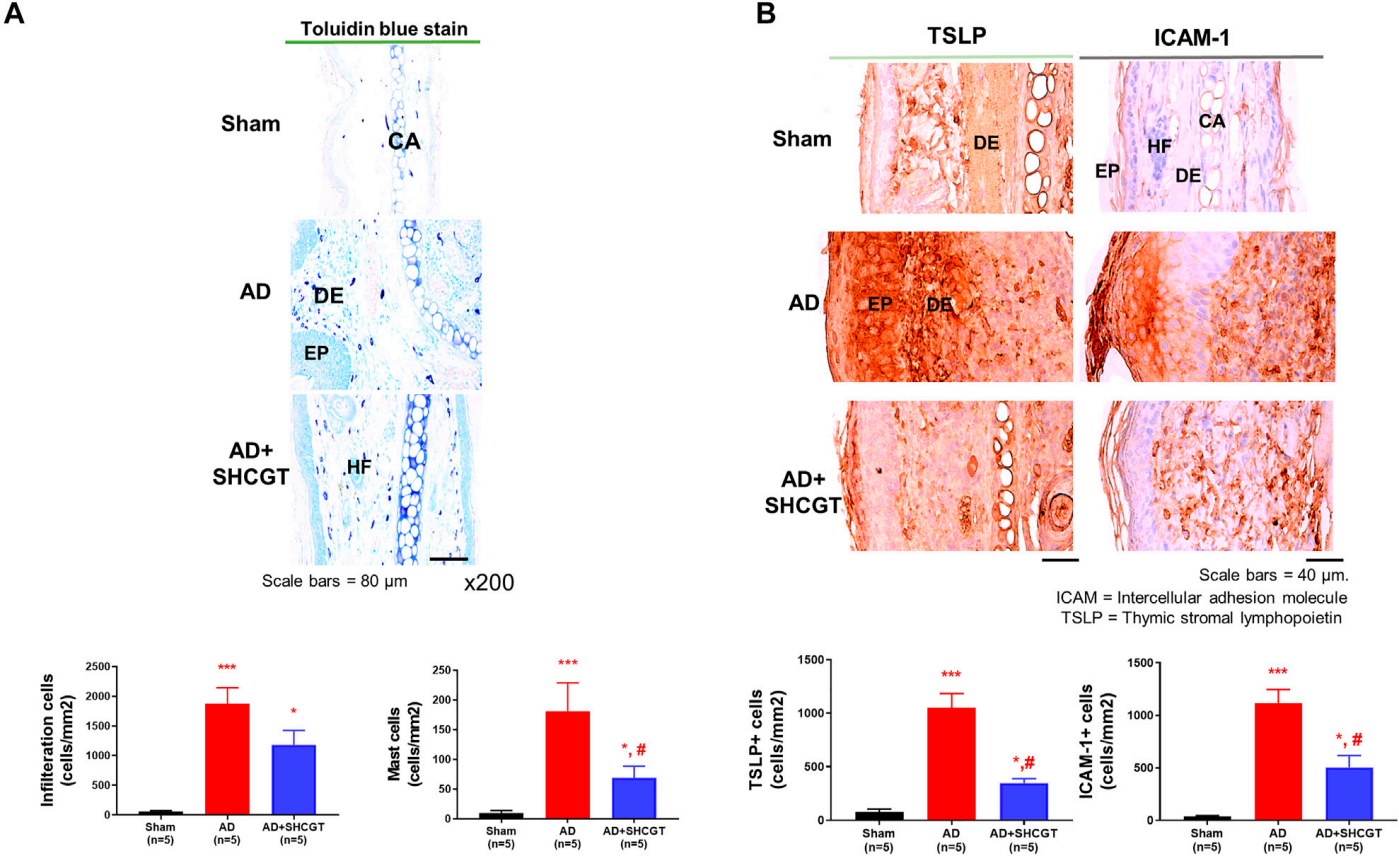
SHCGT inhibited the infiltration of inflammatory cells and the expression of TSLP and ICAM-1 in mice with DNCB-induced AD. **(A)** Toluidine blue-stained mast cells in ear skin tissue. SHCGT reduced the infiltration of inflammatory cells, including mast cells, in the ear skin of mice with DNCB-induced AD. **(B)** Immunohistochemistry staining for TSLP and ICAM-1 in ear skin. SHCGT significantly decreased the expression of TSLP and ICAM-1 in mice with DNCB-induced AD. *p < 0.05, ***p < 0.001 vs. sham; ^#^ *p* < 0.05 vs. AD. AD, atopic dermatitis; DNBC, 2,4-dinitrochlorobenzene; ICAM-1, intercellular adhesion molecule-1; IgE, immunoglobulin E; SHCGT, *Sihocheonggan-Tang*; TSLP, thymic stromal lymphopoietin.

A significantly greater number of cells including mast cells infiltrated the skin in the AD group and the AD + SHCGT group than in the sham group (Figure 3A). There was a 37.9% decrease in the number of mast cells in the AD + SHCGT group compared to the AD group. Furthermore, immunohistochemistry showed that the expression of TSLP-positive and ICAM-1-positive cells in the epidermis and dermis of the ear skin was significantly higher in the AD group and AD + SHCGT group than in the sham group (Figure 3B). Administration of SHCGT decreased the populations of epidermal and dermal TSLP-positive and ICAM-1-positive cells by 54 and 33%, respectively, in the AD group, indicating that SHCGT reduced the inflammatory reaction.

### Effect of SHCGT on the Immune Response in Mice With DNCB-Induced AD

**FIGURE 4:**
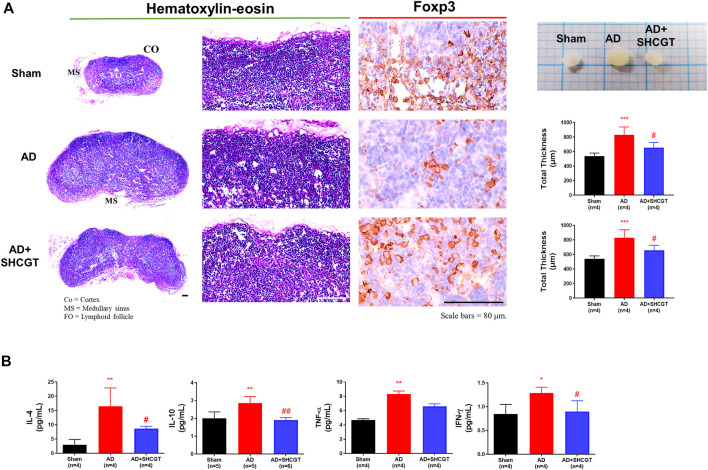
SHCGT suppressed enlargement of peripheral lymph nodes and secretion of cytokines in the spleens of mice with DNCB-induced AD. **(A)** Immunohistochemical lymph node profiles showing that SHCGT reduced the marked hypertrophic changes in the expression of Foxp3, a marker of regulatory T-cell activity, in lymph node tissue from mice with DNCB-induced AD. **(B)** IL-4, IL-10, TNF-α, and IFN-γ levels in the spleen. Increased Th2 (IL-4 and IL-10)-related and Th1 (TNF-α and IFN-γ)-related cytokines were decreased by SHCGT in mice with AD. *p < 0.05, **p < 0.01, ***p < 0.001 vs. sham; ^#^ *p* < 0.05, ^##^ *p* < 0.01 vs. AD. AD, atopic dermatitis; CO, cortex; DNCB, 2,4-dinitrochlorobenzene; FO, lymphoid follicle; ICAM-1, intercellular adhesion molecule-1; IFN-γ, interferon-gamma; IL, interleukin; MS, medullary sinus; SHCGT, *Sihocheonggan-Tang*; TNF-α, tumor necrosis factor-alpha.

We examined the lymph nodes to determine whether topical exposure to DNCB provoked immune activation in mice. DNCB resulted in an increase in lymph node size compared with that of the sham group; this increase was reduced by SHCGT (Figure 4A).

The production of IgE is a characteristic of AD. Increased total IgE levels reflect a switch from Th1 to Th2 (Elghazali et al., 1997). In addition, Foxp3+ Treg cells control the recruitment of inflammatory cells and the expression of Th2 cytokines, as well as elevated serum IgE levels (Fyhrquist et al., 2012). In this study, we also observed marked hypertrophic changes with a decrease in Foxp3+ cells in lymph nodes in the AD group compared with those in the sham group (Figure 4A). However, compared with that of the AD group, the expression of Foxp3+ cells in lymph node tissues was significantly increased in the AD + SHCGT group, and there were changes in Th2 cytokines, including IL-4 and IL-10, in the spleen. IL-4 and IL-10 levels were significantly increased in the AD group and attenuated by administration of SHCGT (Figure 4B).

A surge in Th1 cytokines is characteristic of the chronic and late phases of AD (Chen et al., 2004). In this study, we observed a significant increase in TNF-α and IFN-γ levels in the AD group in comparison with those in the sham group; however, compared with those in the AD group, there was a significant decrease in TNF-α and IFN-γ levels in the AD + SHCGT group (Figure 4B). Furthermore, the decreasing trend of IL-4, IL-10, TNF-α, and INF-γ in the spleen showed a more clearly SHCGT dose-dependent change (Supplementary Results and Supplementary Figure S2).

### SHCGT-Induced Changes in the Gut Microbiome in Mice With DNCB-Induced AD

**FIGURE 5:**
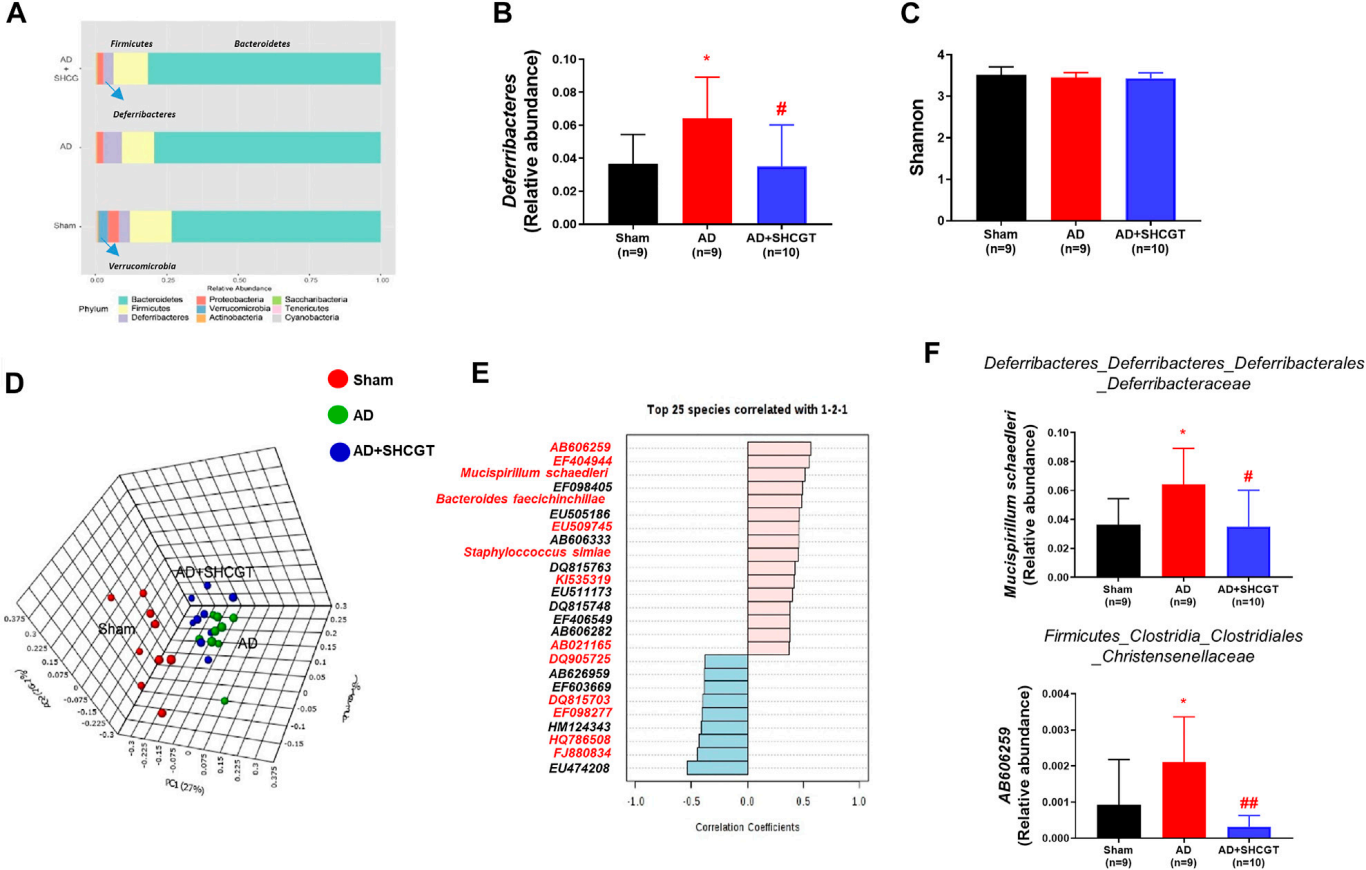
SHCGT changed the gut microbiome in mice with DNCB-induced AD. **(A)** Taxonomic composition of the microbial community in the gut at the phylum level. **(B)** The increased relative abundance of *Deferribacteres* was attenuated by SHCGT in mice with AD. **(C)** Alpha diversity using the Shannon index score at the species level. No significant differences were observed. **(D)** A three-dimensional score plot based on principal coordinates analysis of weighted UniFrac distances showing different microbiome patterns in each group. **(E)** Top 25 species detected in pattern search analysis. The red letters indicate the gut microbiota components also detected by LEfSe analysis. **(F)** A significantly altered gut microbiota profile was identified by a combination of pattern search analysis, LEfSe, and univariate analysis. *p < 0.05 vs. sham; ^#^ *p* < 0.05, ^##^ *p* < 0.01 vs. AD. AD, atopic dermatitis; DNCB, 2,4-dinitrochlorobenzene; LEfSe, linear discriminant analysis effect size; SHCGT, *Sihocheonggan-Tang*.

Recent studies have reported that the gut microbiome plays an important role in AD by regulating metabolites and the immune response (Penders et al., 2007; Song et al., 2016). Therefore, we investigated the changes in the microbiome population after treatment with SHCGT. As shown in Figure 5A, the microbiome in sham mice was dominated by *Bacteroidetes* and *Firmicutes* at the phylum level in terms of relative abundance. *Verrucomicrobia*, which includes *Akkermansia* at the species level, was present in only the sham mice. The relative abundance of *Deferribacteres* was significantly higher in the AD group and was restored by SHCGT (Figure 5B). However, the Shannon index values, which represent community richness and evenness, were not significantly different between the sham, AD, and AD + SHCGT groups (Figure 5C).

A three-dimensional score plot obtained by principal coordinates analysis of weighted UniFrac distances showed different microbiome patterns between the sham, AD, and AD + SHCGT groups (Figure 5D). LEfSe analysis revealed significant alterations in 50 species based on the *p*-value (<0.05) and linear discriminant analysis score (>3; Supplementary Table S2). Based on the results of a pattern search, 17 of these species (*Mucispirillum schaedleri*, *Bacteroides faecichinchillae*, *AB021165*, *EU509745*, *EF098405*, *EU474208*, *EF098277*, *HQ786508*, *FJ880834*, *EF404944*, *KI535319*, *DQ815703*, *EF603669*, *DQ905725*, *AB606259*, *HM124343*, and *Staphylococcus simiae*) showed significant patterns of recovery in the AD + SHCGT group (Figure 5E, Supplementary Figure S3). In particular, the relative abundances of *AB606259* and *M. schaedleri* in the AD group were significantly higher than those in the sham group but improved after treatment with SHCGT (Figure 5F).

### Change in the Intestinal Barrier System and Microbiome Producing SCFAs in Mice With AD After Treatment With SHCGT

Changes in the gut microbiome impaired the intestinal barrier system, leading to inflammatory events involved in the occurrence and development of human AD via changes in microbiota-derived metabolites, including SCFAs (Craig, 2016; Levy et al., 2016).

**FIGURE 6:**
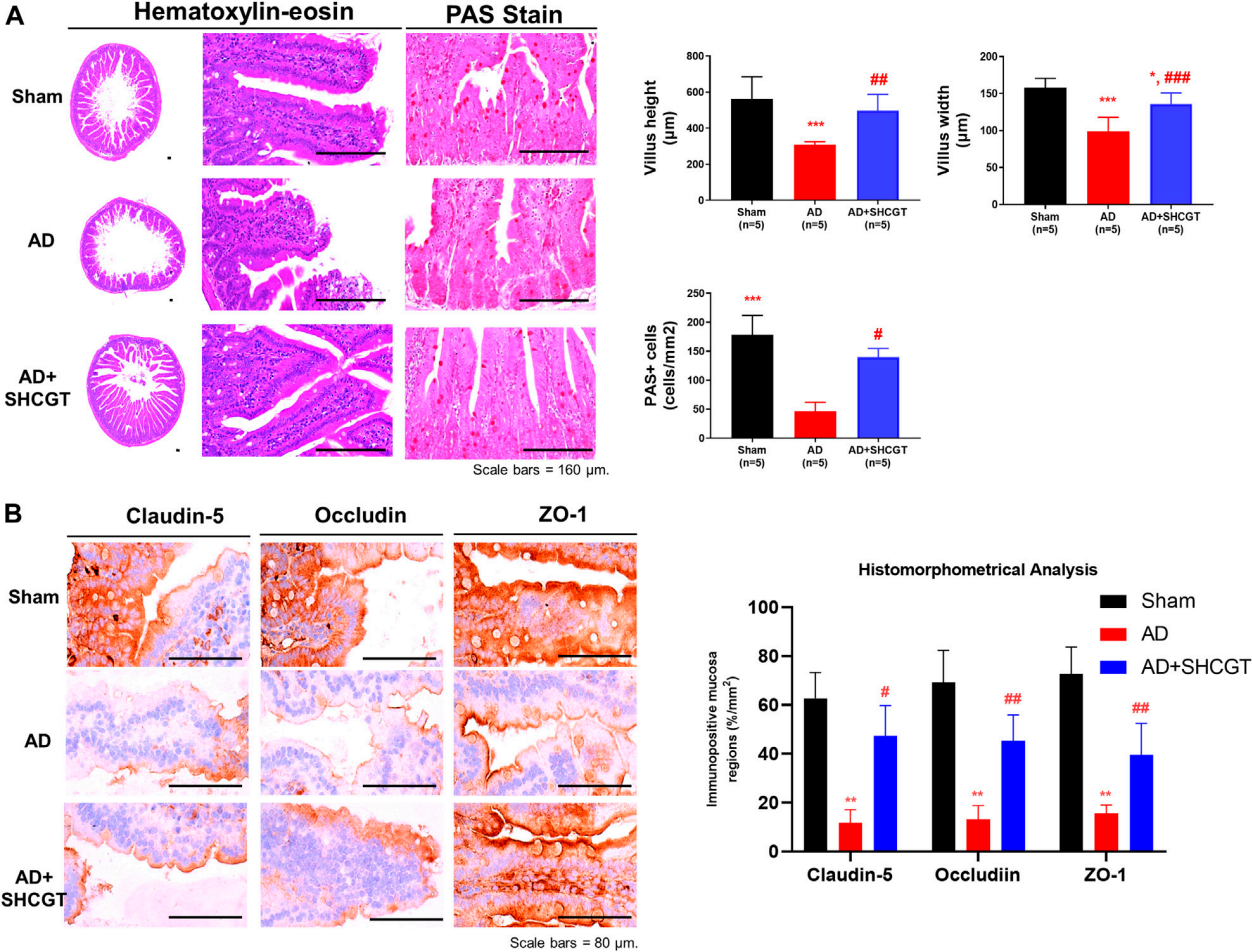
SHCGT improved the damaged intestinal barrier system in mice with DNCB-induced AD. **(A)** Hematoxylin-eosin and PAS staining of intestinal villi. SHCGT significantly reduced the mean villus height and width as well as PAS+ cell numbers in mice with DNCB-induced AD. **(B)** Immunohistochemistry staining for claudin-1, occludin, and ZO-1 in the intestine. Quantitative imaging analysis showed that SHCGT recovered the function of the intestinal barrier system, with losses of tight junctional markers (claudin-5, occludin, and ZO-1). **p < 0.01, ***p < 0.001 vs. sham; ^#^ *p* < 0.05, ^##^ *p* < 0.01, ^###^ *p* < 0.001 vs. AD. AD, atopic dermatitis; DNCB, 2,4-dinitrochlorobenzene; PAS, periodic acid Schiff; SHCGT, *Sihocheonggan-Tang*; ZO-1, zonula occludens-1.

We also observed marked intestinal villous atrophy in the group with DNCB-induced AD in comparison with the sham group (Figure 6A). However, administration of SHCGT caused a significant decrease in mean villus height and width, reduced numbers of PAS-positive cells, and increased total and cortex thicknesses and numbers of cortical lymphoid follicles (Figure 6A). Furthermore, dysfunction of the barrier system, that is, loss of tight junction markers (claudin-5, occludin, and ZO-1), was observed in the group with DNCB-induced AD compared with the sham group (Figure 6B). Similarly, there was significant recovery of mean claudin-5, occludin, and ZO-1+ levels in the mucosal tissues of the small intestine after treatment with SHCGT (Figure 6B). These results suggest that DNCB-induced AD and related immune responses are mediated by hypersensitivity and small intestinal dysfunction and that administration of SHCGT attenuates the loss of tight junctional proteins and related villus atrophy.

**FIGURE 7:**
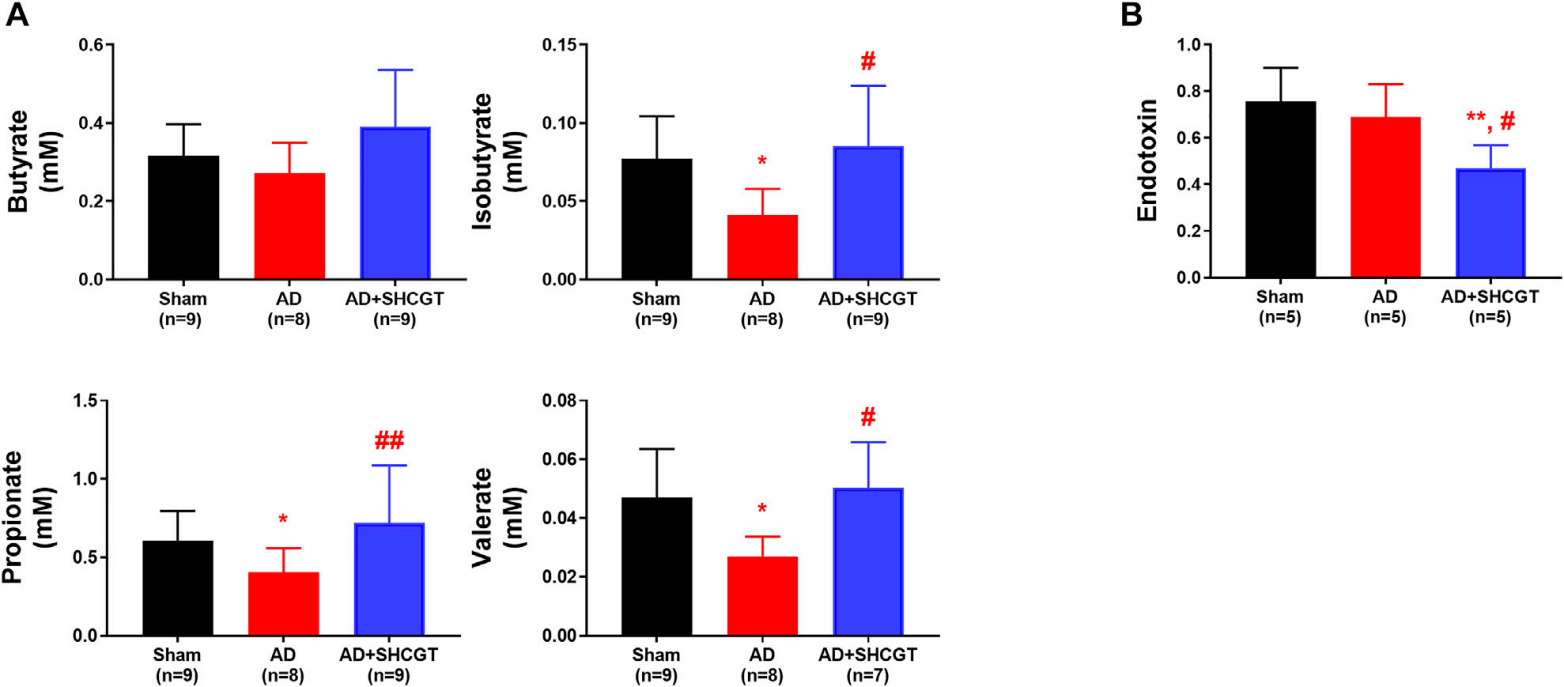
SHCGT regulated gut microbiome-related metabolites and short-chain fatty acids and reduced endotoxin levels. **(A)** Concentrations of short-chain fatty acids, including butyrate, isobutyrate, propionate, and valerate. SHCGT significantly increased the isobutyrate, propionate, and valerate levels in mice with DNCB-induced AD. **(B)** Change in plasma endotoxin levels. The endotoxin level was lower in the group with SHCGT-treated AD than in the sham and AD groups. *p < 0.05, **p < 0.01 vs. sham; ^#^ *p* < 0.05, ^##^ *p* < 0.01 vs. AD. AD, atopic dermatitis; DNCB, 2,4-dinitrochlorobenzene; SHCGT, *Sihocheonggan-Tang*.

Gut microbiota-derived metabolites, including SCFAs, take part in maintaining the integrity of the epithelial barrier and regulating inflammatory responses. As shown in Figure 7, the concentrations of isobutyrate, propionate, and valerate were lower in the mice with DNCB-induced AD than in the sham mice. Administration of SHCGT restored the decreased levels of isobutyrate, propionate, and valerate in the AD group (Figure 7A). However, endotoxin levels in the AD group were not significantly different from those in the sham group but were decreased by SHCGT (Figure 7B). These findings indicate that the reduction in endotoxin levels by SHCGT may contribute to the mitigation of AD, although endotoxins would not be the main cause of AD (Kim and Kim, 2019).

### Effect of SHCGT, Compounds, and SCFAs on TNF-α/IFN-γ-Induced mRNA Expression in HaCaT Cells

Inflammatory keratinocytes are important in the pathogenesis of AD. Exposure of keratinocytes to TNF-α and IFN-γ induces abnormal expression of cytokines and adhesion molecules, such as IL-6 and ICAM-1, which are associated with infiltration of monocytes into skin. To identify other possible effects of microbiota-derived SCFAs on AD, we treated HaCaT cells with isobutyrate, propionate, and valerate.

**FIGURE 8:**
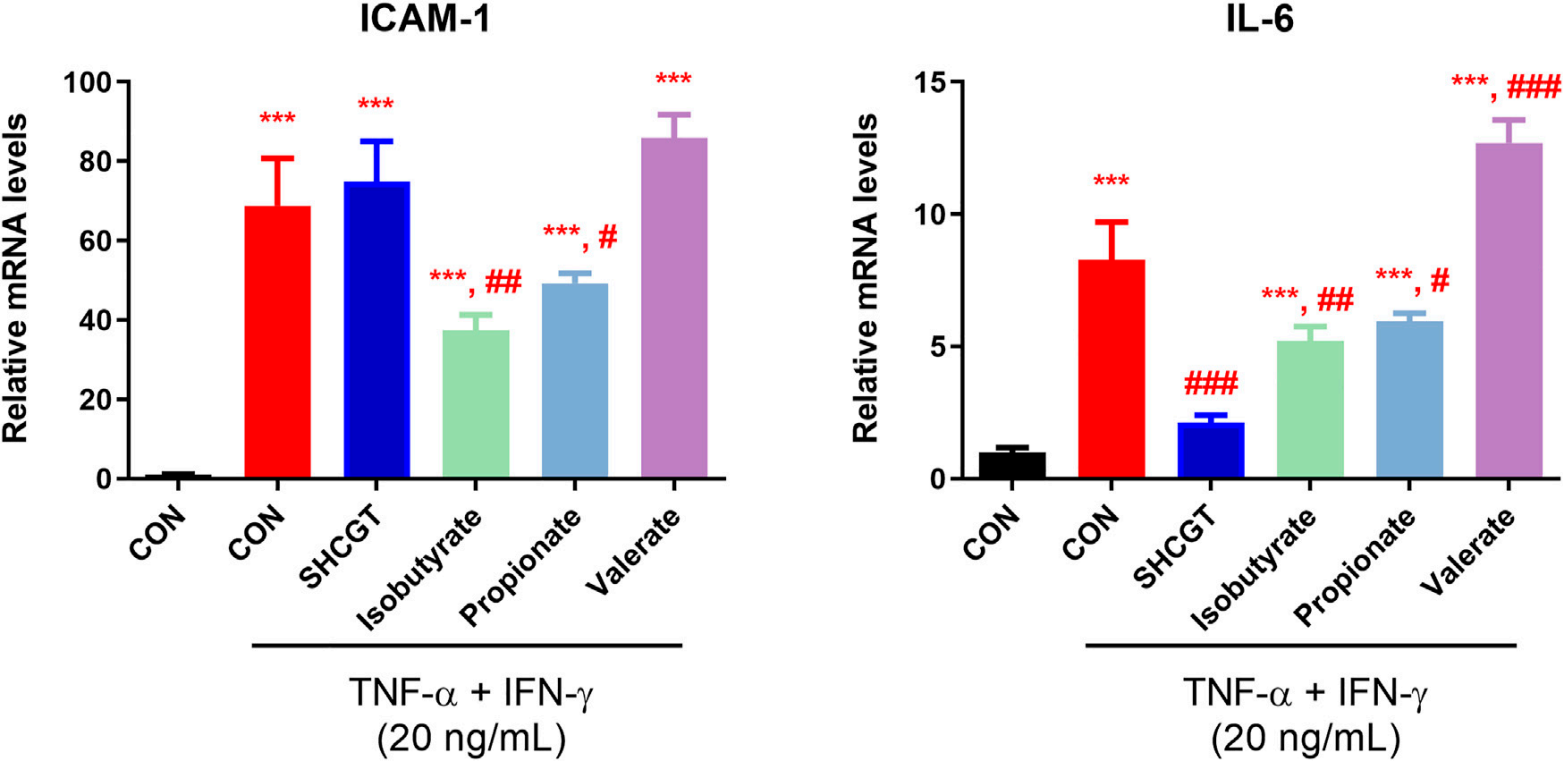
Gut microbiome-related metabolites (short-chain fatty acids) had a beneficial effect on TNF-α/IFN-γ-treated HaCaT cells. Isobutyrate and propionate directly reduced the expression levels of ICAM-1 and IL-6 in TNF-α/IFN-γ-treated HaCaT cells. ***p < 0.001 vs. CON; ^#^ *p* < 0.05, ^##^ *p* < 0.01, ^###^ *p* < 0.001 vs. TNF-α/IFN-γ-treated CON (CON + TNF-α/IFN-γ). AD, atopic dermatitis; CON, control HaCaT cells; DNCB, 2,4-dinitrochlorobenzene; ICAM-1, intercellular adhesion molecule-1; IFN-γ, interferon-gamma; IL-6, interleukin-6; SHCGT, *Sihocheonggan-Tang*; TNF-α, tumor necrosis factor-alpha.

No cytotoxicity was observed in HaCaT cells treated with 1.25–2.5 mM SFCAs (Supplementary Figure S4). As shown in Figure 8, TNF-α/IFN-γ upregulated IL-6 and ICAM-1 expression in HaCaT cells. However, SHCGT directly downregulated the expression of IL-6 but not that of ICAM-1. Instead, treatment with isobutyrate and propionate significantly downregulated TNF-α/IFN-γ-induced IL-6 and ICAM-1 in these cells, suggesting that the gut microbiome-derived metabolites isobutyrate and propionate are involved in the attenuation of inflammation in AD.

**FIGURE 9:**
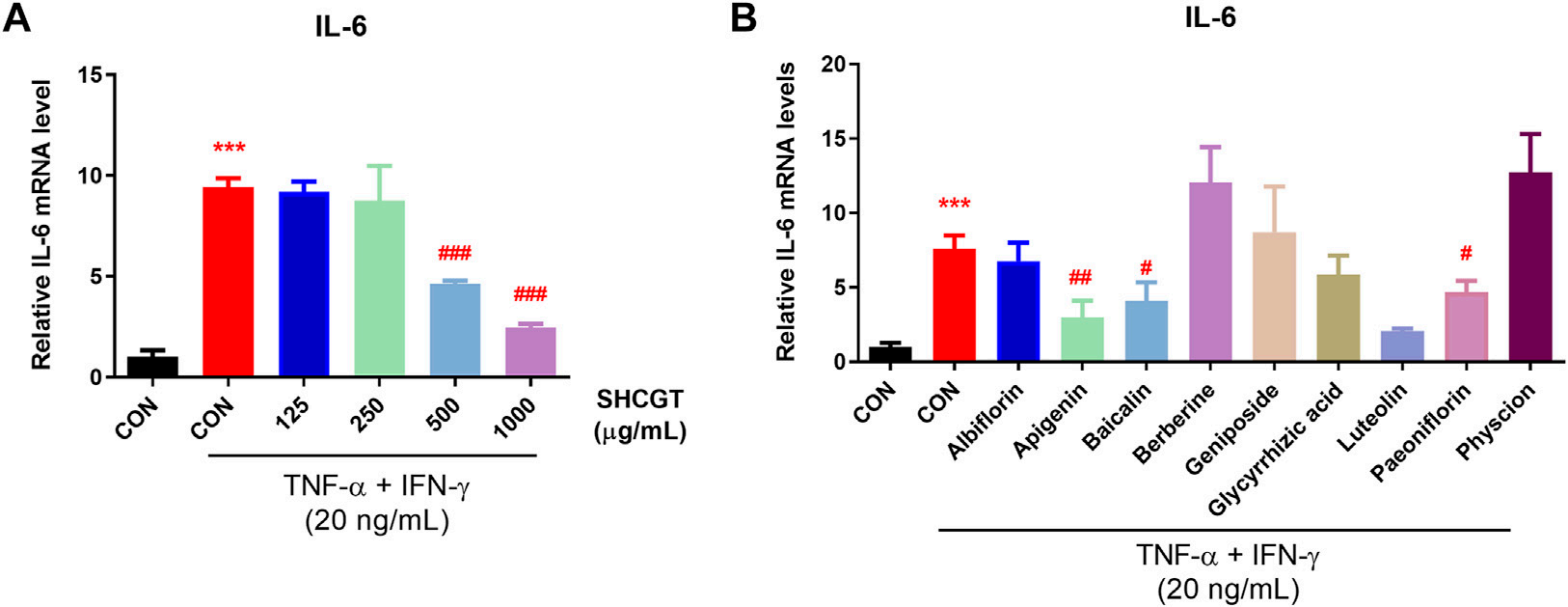
SHCGT dose-dependently affected the atopic dermatitis and bioactive compounds in SHCGT had an inhibitory effect in TNF-α/IFN-γ-treated HaCaT cells. **(A)** SHCGT regulated the expression of IL-6 mRNA expression in TNF-α/IFN-γ-treated HaCaT cells in dose-dependent manners. **(B)** The compounds in SHCGT showed inhibitory effect on IL-6 mRNA expression in TNF-α/IFN-γ-treated HaCaT cells. HaCaT cells were pretreated with SHCGT or compounds at predetermined concentrations (SHCGT, 125–1,000 μg/ml; albiflorin, 100 μM; baicalin, 50 μM; geniposide, 100 μM; glycyrrhizic acid, 100 μM; paeoniflorin, 100 μM; apigenin, 25 μM; luteolin, 25 μM; berberine hydrochloride, 50 μM; and physcion, 100 μM) for 6 h and then stimulated with TNF-α (20 ng/ml) and IFN-γ (20 ng/ml) for an additional 18 h. ***p < 0.001 vs. CON; ^#^ *p* < 0.05, ^##^ *p* < 0.01, ^###^ *p* < 0.001 vs. TNF-α/IFN-γ-treated CON (CON + TNF-α/IFN-γ). CON, control; TNF-α, tumor necrosis factor-alpha; IFN-γ, interferon-gamma; IL-6, interleukin-6; SHCGT, *Sihocheonggan-Tang*.

Furthermore, we examined the mRNA expression of IL-6 in TNF-α/IFN-γ-induced HaCaT cells to evaluate whether SHCGT dose-dependently affects the atopic dermatitis. No cytotoxicity was observed with SHCGT up to 1,000 μg/ml in HaCaT cells (Supplementary Figure S4). However, SHCGT dose-dependently decreased the TNF-α/IFN-γ-induced mRNA expression of IL-6 in HaCaT cells (Figure 9A). In addition, the detailed chemical investigation was done to predict the most active compounds responsible for its anti-atopic dermatitis effect. We determined the concentration of each compound, not having cytotoxicity by CCK-8 assay (Supplementary Figure S4). Albiflorin (100 μM), baicalin (50 μM), geniposide (100 μM), glycyrrhizic acid (100 μM), paeoniflorin (100 μM), apigenin (25 μM), luteolin (25 μM), berberine hydrochloride (50 μM), and physcion (100 μM) were used to confirm the inhibitory effect of IL-6 mRNA expression in HaCaT cells. The results showed that apigenin, baicalin, luteolin, and paeoniflorin could be associated with anti-atopic dermatitis effect of SHCGT (Figure 9B).

Taken all results together, we suggested that SHCGT relieves the atopic dermatitis in a dose-dependent manner, and SCFAs produced by intestinal microorganisms changed by SHCGT also play a role in improving the atopic dermatitis.

## Discussion

Topical steroids and oral antihistamines are the first-line treatment for AS (Eichenfield et al., 2014). However, these agents have several adverse effects, and many patients with AD are reluctant to use steroids in the long term (Kojima et al., 2013). Therefore, there is increasing interest in herbal medicines as a treatment for AD worldwide, given that many herbs and traditional Chinese medicines are commonly used to treat allergic disorders, including AD, asthma, and arthritis (Ma et al., 2013). Furthermore, several studies have investigated the potential role of herbal medicines as prebiotics for modulation of the growth and activity of microorganisms that play a crucial role in metabolism and the immune response in various diseases (Hutkins et al., 2016; Lee et al., 2020).

SHCGT is an herbal medicine that has been used to treat AD in Korea (An et al., 2017). However, detailed mechanistic studies in animal models have not been reported. In this study, we demonstrated the pharmacological effects of SHCGT in a mouse model of DNBC-induced AD and investigated the ability of SHCGT to regulate the gut microbiome in mice with AD.

Several studies have suggested that abnormalities in the composition and function of the gut microbiome contribute substantially to the onset of AD and atopic march (Kim and Kim, 2019). Watanabe et al. (2003) reported a significantly lower *Bifidobacterium* count and a significantly higher *Staphylococcus* count in patients with AD than in healthy controls and that the levels of these microorganisms differed according to disease state. Interestingly, in our study, we found that mice with AD had increased levels of *S. simiae*, a coagulase-negative bacterium that is closely related to *S. aureus*, which is frequently isolated from the skin in patients with AD during flares (Suzuki et al., 2012; Geoghegan et al., 2018).

Moreover, Song et al. (2016) showed that enrichment of subspecies of *Faecalibacterium prausnitzii* (F06) decreased high butyrate and propionate producers, contributing to impairment of the gut epithelial barrier in patients with AD. In particular, they suggested that damaged epithelium with increased permeability might allow systemic circulation of undigested foods, toxins, and pathogenic microorganisms, ultimately leading to an aberrant Th2-type immune response in the skin of these patients (Pike et al., 1986; Noverr and Huffnagle, 2004). In the present study, treatment with SHCGT significantly decreased the endotoxin level in the AD group, but there was no significant difference in the endotoxin level between the sham and AD groups, indicating that endotoxins may not be the main cause of AD.

SHCGT-induced attenuation of damage to the intestinal barrier system in mice with AD was accompanied by a change in the composition of the gut microbiome, including in *AB606259* and *M. schaedleri*. *M. schaedleri* is found in abundance in the mucosal layer of the intestine. This organism has been suggested to be a pathobiont that modifies gene expression in the host mucosal tissue and has proinflammatory properties that affect inflammation status or susceptibility to disease (Loy et al., 2017). *M. schaedleri* has limited ability to degrade complex polysaccharides and uses monosaccharides, oligopeptides, amino acids, glycerol, and SCFAs, that is, the breakdown products of other members of the microbiota, as substrates for energy metabolism (El Kaoutari et al., 2013; Loy et al., 2017). In accordance with these properties, we found that the levels of SCFAs, including isobutyrate, propionate, and valerate, were significantly lower in mice with AD than in sham mice and returned to the sham levels after treatment with SHCGT.

SCFAs, which are derived from the degradation of polysaccharides by the gut microbiome, participate in the proliferation and differentiation of both B-cells and T-cells and have anti-inflammatory effects (Nguyen et al., 2016; Kim and Kim, 2019). In this study, we found that SHCGT suppressed the increased Th2 and Th1 immune responses in mice with AD and that SCFAs also directly reduced the expression of IL-6 and ICAM-1 in TNF-α/INF-γ-induced keratinocytes. SHCGT contains several compounds that are known to attenuate AD, including berberine and paeoniflorin (Tsang et al., 2016; Jo et al., 2018), and was also found to decrease the expression of IL-6 in an *in vitro* model of AD in our study. However, the actual concentrations of these active compounds in SHCGT may be extremely low in the circulation after oral administration. Therefore, it is possible that SHCGT influences the gut microbiome and that the polysaccharides and indigestible fibers in SHCGT could act as prebiotics by supplying SCFAs to the host circulation (Peterson et al., 2018).

## Conclusion

In this study, we found that oral administration of SHCGT ameliorated AD-like symptoms and modulated the composition of the gut microbiota, with beneficial effects on AD. However, further studies are necessary to determine the crucial component of SHCGT that controls intestinal bacteria and to identify the underlying mechanism of the effect of the microbiota on AD. The dose of SHCGT was determined by considering the dose used in clinical practice, but it was much higher than the concentration given in a consensus document (Heinrich et al., 2020). Therefore, more experiments with appropriate concentrations are needed to optimize the dosage and explore the effect of SHCGT. Nevertheless, findings in this study advance our knowledge about SHCGT and successfully demonstrate the potential role of herbal medicines as prebiotics for the management of AD.

## Data Availability

The datasets presented in this study can be found in online repositories. The names of the repository/repositories and accession number(s) can be found below: https://www.ncbi.nlm.nih.gov/, PRJNA739837.
