# Supplementary material for: Modulation of the Gut Microbiota by Sihocheonggan-Tang Shapes the Immune Responses of Atopic Dermatitis
Source: Front Pharmacol. 2021 Sep 20;12:722730. doi: 10.3389/fphar.2021.722730 (PMC8489559; doi:10.3389/fphar.2021.722730)
Supplement: Supplementary file 1 [file DataSheet1.docx]

**Modulation of the Gut Microbiota by *Sihocheonggan*-*Tang* Shapes the Immune Responses of Atopic Dermatitis**

**Jaemoo Chun^1#^, So Min Lee^2#^, You Mee Ahn^1^, Min-gyung Baek^3,4^, Hana Yi^5^, Shin Sarah^1^, Jeeyoun Jung^1^***

^1^ KM Science Research Division, Korea Institute of Oriental Medicine, Daejeon 34054, Korea

^2^ KM Convergence Research Division, Korea Institute of Oriental Medicine, Daejeon 34054, Korea

^3^Interdisciplinary Program in Precision Public Health, Korea University, Seoul 02841, Korea

^4^Department of Public Health Sciences, Korea University, Seoul 02841, Korea

^5^School of Biosystems and Biomedical Sciences, Korea University, Seoul 02841, Korea

*** Correspondence:**Jeeyoun Jung
jjy0918@kiom.re.kr

# These authors contributed equally to this work

**Supplementary Methods**

***Preparation of the SHCG-tang***

SHCGT was purchased from Hanpoong Pharm & Foods Co. Ltd. (Jeonju, Republic of Korea). The SHCG-tang was prepared by extracting a mixture of fifteen types of dried medicinal herbs, which included the following: 0.67 g of Bupleuri radix, 0.5 g of Forsythiae Fructus, 0.5 g of Cnidium officinale, 0.5 g of Trichosanthis Radix, 0.5 g of Scutellariae Radix, 0.5 g of Gardeniae Fructus, 0.5 g of Paeoniae Radix, 0.5 g of Arctii Fructus, 0.5 g of Coptidis Rhizoma, 0.5 g of Angelicae Gigantis Radix, 0.5 g of Platycodi Radix, 0.5 g of Rehmanniae Radix Preparat, 0.5 g of Menthae Herba, 0.5 g of Phellodendri Cortex, 0.5 g of Glycyrrhizae Radix. Briefly, The SHCGT comprising fifteen herbal medicines was mixed and extracted in a 10-fold mass in distilled water at 100°C for 2 h. The aqueous extract was filtered by pressing through a filter (10 μm pore size), and then the solution was evaporated and freeze-dried to give a powder. The amount of SHCGT powder was 1.32 g (extract yield: 17.2%), which was then stored at 4˚C until use. The details about species of fifteen herbal medicines were described in **Supplementary Table 1.**

***UPLC/Q-TOF MS Analysis of SHCG-tang***

We performed UPLC (Waters Corp., Milford, MS, USA) combined with Q-TOF MS (impact HD; Bruker, Bremen, Germany) analysis to verify the chemical components of the SHCG-tang (Fig. S1). The power of SHCGT (10 mg) was mixed with 1 mL methanol-water solution (50:50, *v/v*) was added. The sample was vortexed for 1 min and then centrifuged at 12,500 *g* and 25°C for 20 min. The supernatant was collected and filtered using a Millex®-LG filter with a 0.20 μm pore size (Millipore, Billerica, MA, USA). A UPLC BEH C18 column (100$\times$ 2.1 mm, 1.7 μm particle size; Waters Corp., Milford, MA, USA) was used to separate the SHCGT at 40°C. The mobile phase consisted of water with 0.1% (*v/v*) formic acid (solvent A) and acetonitrile with 0.1% (*v/v*) formic acid (solvent B). The flow rate was set to 0.45 mL/min for the total 20 min run time. The linear gradient of UPLC was as follows: 5% B for 2 min, 5% to 30% B from 2 min to 10 min, 30% to 50% B from 10 min to 14 min, 50% to 99% B from 14 min to 16 min, 99% B from 16 min to 18 min, 5% B from 18 min to 20 min. The injection volume of each standard or SHCGT sample was 3 μL. The mass spectrometer was operated in positive ionization mode with a mass range of *m/z* 50 to 1000 at a sampling rate of 3 Hz. The MS parameters were set as follows: capillary voltage, +4500 V; end plate offset, 500 V; nebulizer gas, 1.5 bar; dry gas, 5 L/min at 250°C. Nitrogen was used as the drying, nebulizing, and collision gas. The MS/MS analyses were acquired by automatic fragmentation with a cycle time of 3 s.

The UPLC/Q-TOF MS data were processed using MS-Dial and also identified chemical components of the SHCGT based on the retention time, m/z, and the MS fragment pattern using in all publicly available mass spectral databases obtained from RIKEN (http://prime.psc.riken.jp/Metabolomics_Software/MS-DIAL/) which includes MS/MS records of MassBank, ReSpect, GNP, Fiehn HILIC, CASMI2016, RIKEN PlaSMA authentic standards, RIKEN PlaSMA bio-MS/MS [1]

**Supplementary Results**

**SHCGT alleviated the atopic dermatitis in dose-dependent manners**

To investigate the dose-dependent effect of SHCGT on AD, we conducted additional experiments (#21-072). DNCB was regularly applied to the dorsal skin of BALB/c mice, and low (313 mg/kg) and high dose (625 mg/kg) of SHCGT were administered orally daily for 2 weeks [Sham (n=5), AD (n=5), AD+SHCGT_Low dose (Low-dose SHCGT treated AD mice, n=5), AD+SHCGT_High dose (High-dose SHCGT treated AD mice, n=5)]. On days 7–21, atopic dermatitis index (ADI) scores were significantly higher in BALB/c mice treated with DNCB than in sham mice (**Supplementary Figure 2A**). On days 14-21, ADI indices were significantly reduced in the low-dose, and high-dose SHCGT treated AD groups (**Supplementary Figure 2A**).

The percentage of neutrophils was significantly higher in AD group than those in Sham group, whereas high dose of SHCGT treated AD group (p=0.07) showed the decrease tendency compared to AD group (**Supplementary Figure 2A**). The IgE levels were also significantly lower in the low-dose, and high dose of SHCGT treated AD groups than in AD group (**Supplementary Figure 2A**). In addition, SHCGT decreased the corticosterone levels of AD mice in dose-dependent manners (**Supplementary Figure 2A)**.

Furthermore, the decreasing trend of Th2 and Th1 cytokines, including IL-4, IL-10, TNF-α, and INF-γ in the spleen, showed a more clearly SHCGT dose-dependent change (**Supplementary Figure 2B**). The levels of IL-10, TNF-α, and INF-γ of AD mice were significantly increased compared to those of Sham mice. The low-dose of SHCGT significantly decreased the IL-4 and TNF-α compared to AD mice, whereas the high-dose of SHCGT significantly reduced the IL-4, IL-10 and TNF-α in AD mice (**Supplementary Figure 2B**). In addition, there were no significant differences in INF-γ between Sham and high-dose SHCGT treated AD mice, whereas AD and low-dose SHCGT treated AD mice had significantly higher levels of INF-γ than the Sham mice (**Supplementary Figure 2B)**.


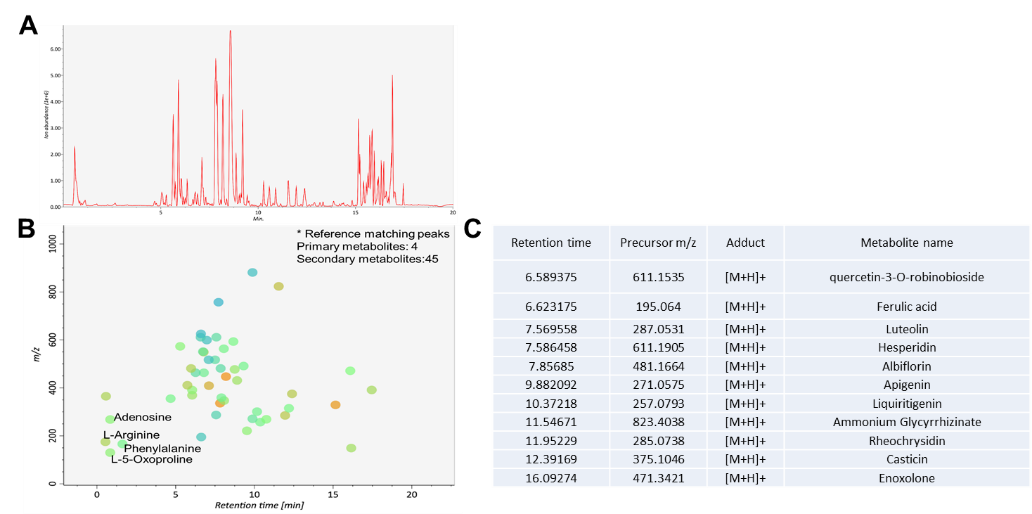


**Figure S1.** Chemical components in SHCGT. The chemical components of SHCGT were identified by UPLC-MS analysis. (A) ESI+ base peak chromatography (BPC) from UPLC-MS chromatography. (B) Peak spot graph representing identified metabolites. (C) List of secondary metabolites


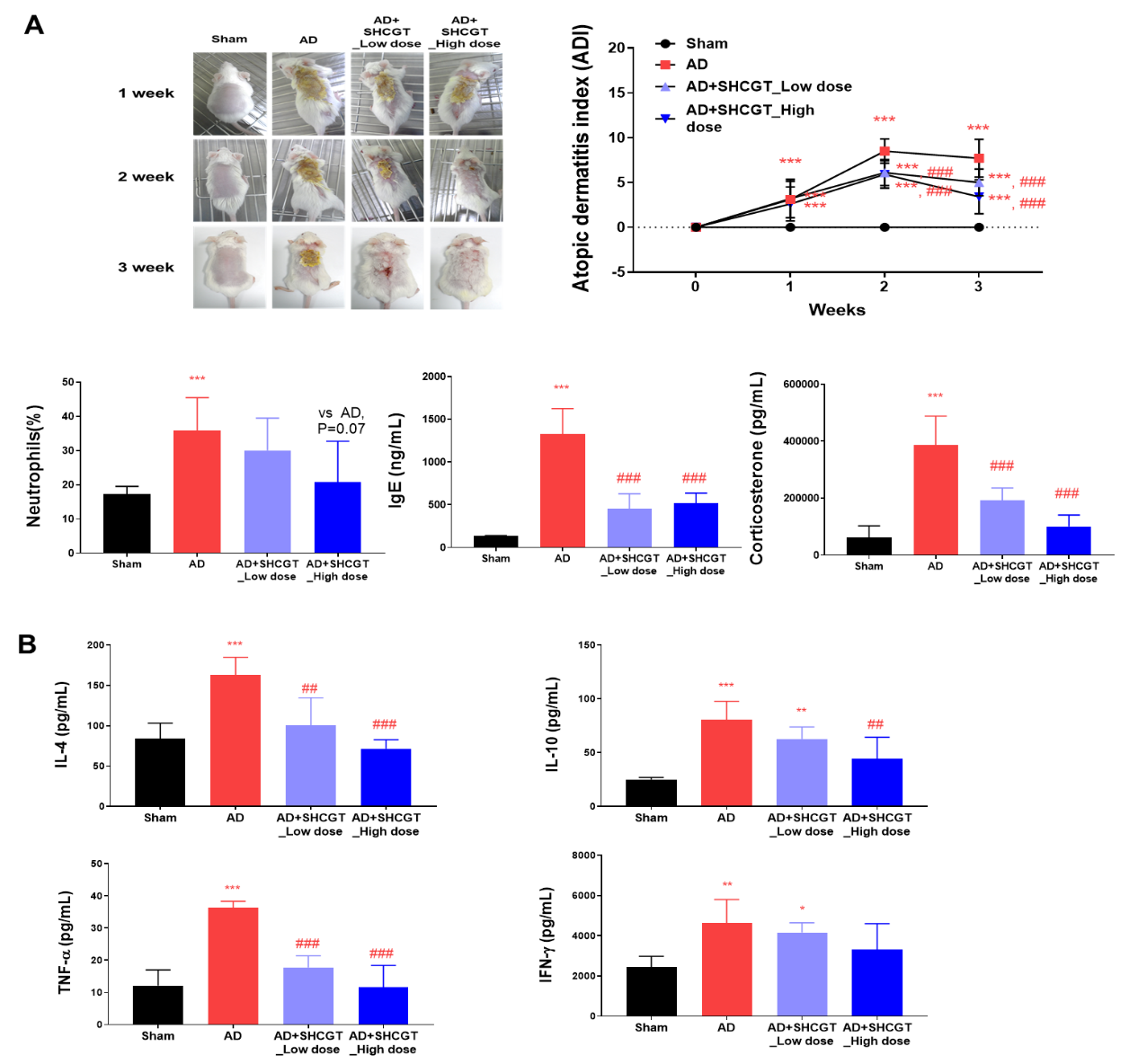


**Figure S2.** SHCGT alleviated the atopic dermatitis in dose-dependent manners. DNCB-induced AD mice were treated with low (313 mg/kg) and high (625 mg/kg) dose SHCGT. (A) The change in atopic dermatitis index (ADI) and levels of IgE and corticosterone were significantly decreased in low and high dose SHCGT treated AD groups, compared to those of AD mice. In particular, the ADI index, the percentage of neutrophil and the levels of corticosterone were changed in dose-dependent manners. (B) SHCGT dose-dependently inhibited the cytokines secretion in the spleens of mice with DNCB-induced AD. IL-4, IL-10, TNF-α, and IFN-γ levels in the spleen. *p<0.05, **p<0.01, ***p<0.001 vs. sham; ^##^p<0.01, ^###^p<0.001 vs. AD. AD, atopic dermatitis; SHCGT, *Sihocheonggan-Tang;* AD+SHCGT_L, low dose SHCGT treated AD group; AD+SHCGT_H, high dose SHCGT treated AD group.


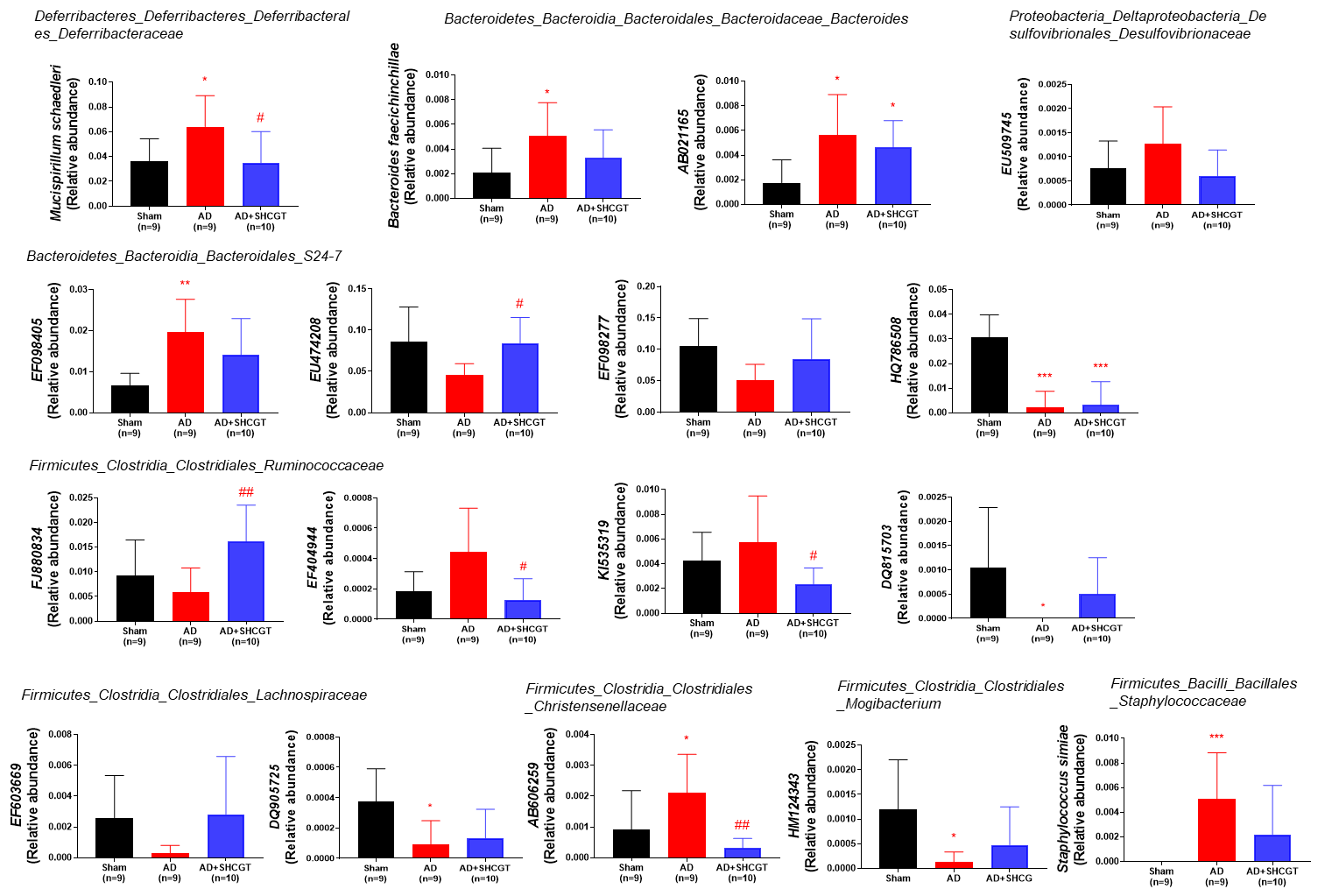


**Figure S3.** Microbiota identiﬁed by pattern search.


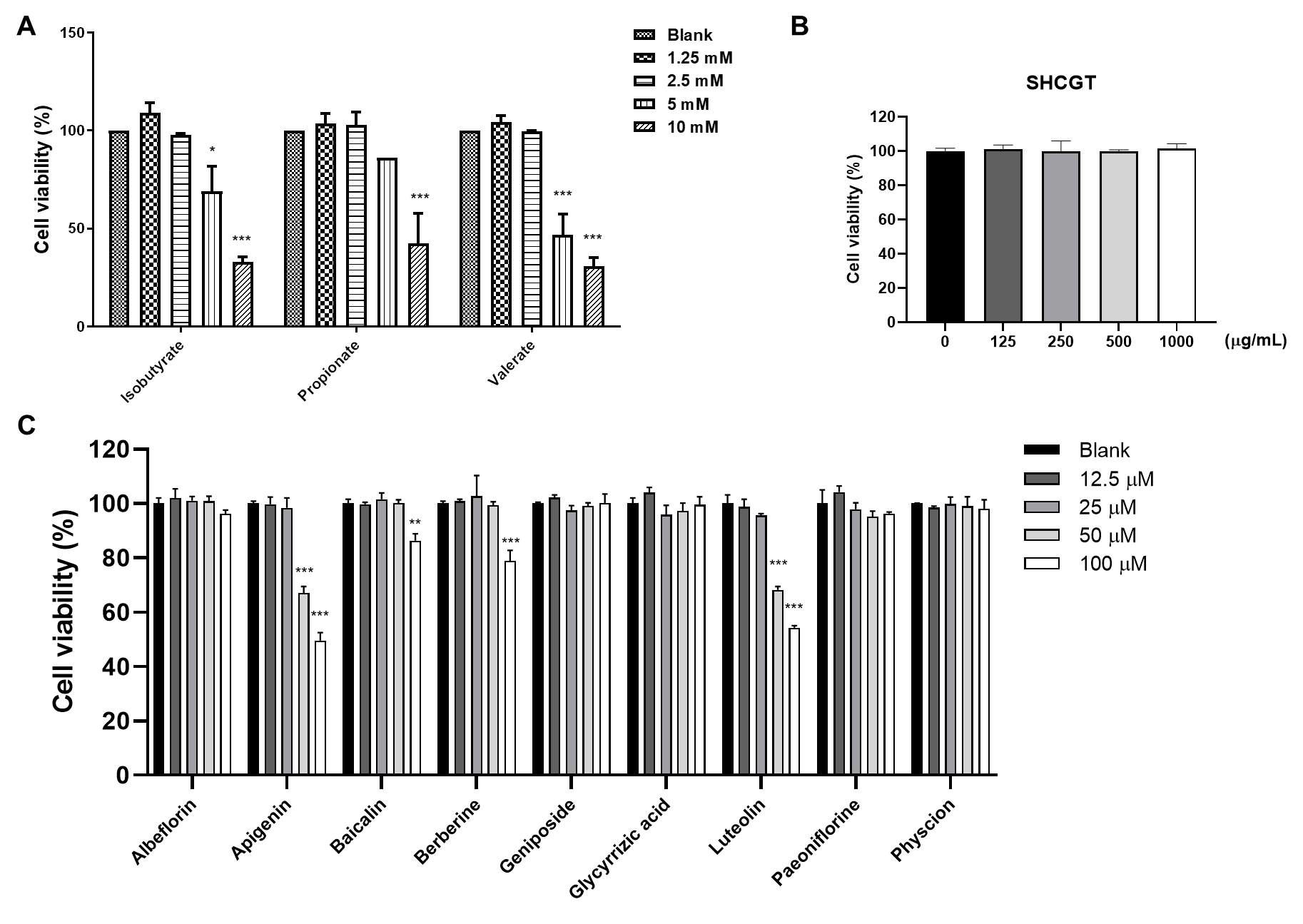


**Figure S4.** Cytotoxicity of **(A)** SCFA, **(B)** SHCGT and **(C)** compounds including albiflorin, baicalin, geniposide, glycyrrhizic acid, paeoniflorin, apigenin, luteolin, berberine hydrochloride, and physcion in HaCaT cells. Cell viability was analyzed using a cell counting kit-8 (CCK-8) assay. SCFA, Short-chain fatty acids; SHCGT, *Sihocheonggan-Tang*

**Table S1.** The composition of *Sihocheonggan*-*Tang*.

| Composition | Family | Scientific name | Amount (g) | Production region |
| --- | --- | --- | --- | --- |
| Bupleuri Radix | Apiaceae | *Bupleurum falcatum* L. | 0.67 | China |
| Forsythiae Fructus | Oleaceae | *Forsythia suspensa* (Thunb.) Vahl | 0.5 | China |
| Cnidii Rhizoma | Umbelliferae | *Ligusticum striatum* DC. | 0.5 | China |
| Trichosanthis Radix | Cucurbitaceae | *Trichosanthes kirilowii* Maxim. | 0.5 | China |
| Scutellariae Radix | Lamiaceae | *Scutellaria baicalensis* Georgi | 0.5 | China |
| Gardeniae Fructus | Rubiaceae | *Gardenia jasminoides* J.Ellis | 0.5 | China |
| Paeoniae Radix | Paeoniaceae | *Paeonia lactiflora* Pall. | 0.5 | China |
| Arctii Fructus | Asteraceae | *Arctium lappa* L. | 0.5 | China |
| Coptidis Rhizoma | Ranunculaceae | *Coptis chinensis* Franch. | 0.5 | China |
| Angelicae Gigantis Radix | Apiaceae | *Angelica gigas*Nakai | 0.5 | China |
| Platycodi Radix | Campanulaceae | *Platycodon grandiflorus* (Jacq.) A.DC. | 0.5 | China |
| Rehmanniae Radix Preparata | Scrophulariaceae | *Rehmannia glutinosa* (Gaertn.) DC. | 0.5 | China |
| Menthae Herba | Lamiaceae | *Mentha arvensis* L. | 0.5 | China |
| Phellodendri Cortex | Rutaceae | *Phellodendron chinense* C.K.Schneid. | 0.5 | China |
| Glycyrrhizae Radix | Fabaceae | *Glycyrrhiza uralensis* Fisch. | 0.5 | Kazakhstan |

**Table S2.** Top 50 features identiﬁed by linear discriminant analysis of effect size (LEfSe)

| Features | P values | Sham | AD | AD+SHCG | LDA score |
| --- | --- | --- | --- | --- | --- |
| *HQ786508* | 4.92E-05 | 317720 | 21012 | 31757 | 5.17 |
| *DQ815395* | 0.000114 | 83831 | 178170 | 272770 | 4.98 |
| *Staphylococcusimiae* | 0.000351 | 0 | 48908 | 23932 | 4.39 |
| *EF098405* | 0.001579 | 68761 | 196280 | 143400 | 4.8 |
| *DQ394637* | 0.001683 | 20867 | 17897 | 1239 | 3.99 |
| *EU622736* | 0.001941 | 14636 | 1159.3 | 326.05 | 3.85 |
| *EF406686* | 0.002673 | 192440 | 453210 | 447150 | 5.12 |
| *EU510197* | 0.003061 | 1738.9 | 5071.9 | 6390.6 | 3.37 |
| *AB606259* | 0.004089 | 9129.4 | 20795 | 3130.1 | 3.95 |
| *AB021165* | 0.004377 | 16665 | 56878 | 47669 | 4.3 |
| *Eubacterium ventriosum* | 0.004987 | 14853 | 4999.5 | 4043 | 3.73 |
| *EU510350* | 0.005277 | 26157 | 14853 | 1891.1 | 4.08 |
| *EU939413* | 0.005766 | 15143 | 869.47 | 260.84 | 3.87 |
| *CDPR* | 0.005985 | 37387 | 78252 | 68993 | 4.31 |
| *Bacteroides uniformis* | 0.006078 | 5868.9 | 17897 | 22172 | 3.91 |
| *EF603964* | 0.006566 | 8839.6 | 1666.5 | 3390.9 | 3.55 |
| *EU474208* | 0.008189 | 891710 | 469150 | 864170 | 5.32 |
| *KI535319* | 0.008199 | 44343 | 58037 | 22889 | 4.24 |
| *HM124343* | 0.00841 | 12825 | 869.47 | 4629.9 | 3 |
| *FJ880834* | 0.008524 | 92671 | 57892 | 167260 | 4.74 |
| *HM124060* | 0.012892 | 54269 | 1014.4 | 16368 | 4.43 |
| *Anaerostipes butyraticus* | 0.013066 | 869.47 | 3115.6 | 5086.4 | 3.32 |
| *FJ879877* | 0.014748 | 3405.4 | 1159.3 | 65.21 | 3.22 |
| *DQ905725* | 0.016405 | 4130 | 652.1 | 1499.8 | 3.24 |
| *JQ083832* | 0.016605 | 2028.8 | 11158 | 6977.5 | 3.66 |
| *Parabacteroides goldsteinii* | 0.017427 | 13912 | 29490 | 39387 | 4.11 |
| *DQ815703* | 0.01761 | 9926.5 | 0 | 4825.6 | 3.7 |
| *GU302582* | 0.017642 | 1883.9 | 1738.9 | 326.05 | 2.89 |
| *EF406567* | 0.017741 | 4709.6 | 1738.9 | 0 | 3.37 |
| *Bacteroides faecichinchillae* | 0.020872 | 18983 | 54994 | 33257 | 4.26 |
| *Akkermansia muciniphila* | 0.022509 | 340760 | 1376.7 | 5216.8 | 5.23 |
| *AB606295* | 0.023279 | 25867 | 5868.9 | 20215 | 4 |
| *Mucispirillum chaedleri* | 0.025836 | 383580 | 651090 | 357550 | 5.17 |
| *AB606386* | 0.026159 | 5506.6 | 3260.5 | 652.1 | 3.39 |
| *FJ880207* | 0.026947 | 6593.5 | 2246.1 | 3586.6 | 3.34 |
| *KE159600* | 0.029891 | 6086.3 | 23693 | 33583 | 4.14 |
| *EF404944* | 0.030527 | 1521.6 | 5216.8 | 1630.3 | 3.27 |
| *FJ881243* | 0.036105 | 37677 | 19491 | 15259 | 4.05 |
| *EF404684* | 0.036563 | 16882 | 12390 | 4434.3 | 3.79 |
| *EF603669* | 0.036995 | 25722 | 3550.3 | 27127 | 4.07 |
| *HM124077* | 0.037259 | 111070 | 31881 | 50929 | 4.6 |
| *PAC000688* | 0.038012 | 18114 | 5579.1 | 5151.6 | 3.81 |
| *EF096213* | 0.038172 | 79991 | 22461 | 42126 | 4.46 |
| *EF603095* | 0.038227 | 10868 | 144.91 | 18389 | 3.96 |
| *EF098277* | 0.038711 | 1099900 | 522550 | 850600 | 5.46 |
| *DQ815942* | 0.039573 | 339310 | 343730 | 507860 | 4.93 |
| *FJ880976* | 0.041816 | 652.1 | 2898.2 | 2608.4 | 3.05 |
| *EF097039* | 0.042751 | 37025 | 16882 | 18455 | 4 |
| *EU509745* | 0.046726 | 8332.4 | 13115 | 5282 | 3.59 |
| *EF604622* | 0.048876 | 60428 | 41590 | 78057 | 4.26 |

**Table S3.** Short chain fatty acids assignment by NMR spectroscopy.

| Compound | δ ^1^H | Multiplicity | Confirmation |
| --- | --- | --- | --- |
| Butyrate | 0.88 | t | 1D, HMQC, TOCSY |
| Propionate | 1.04 | t, dd | 1D, HMQC, TOCSY |
| Isobutyrate | 1.05 | d | 1D, HMQC, Spiking |
| Valerate | 1,51 | m | 1D, HMQC, TOCSY |
| Acetate | 1.90 | s | 1D, HMQC, Spiking |

^1^H chemical shift data for metabolites of fecal samples, showing compound name, chemical group, chemical shift in parts per million (ppm), multiplicity and types of spectra. Abbreviations: HMQC, heteronuclear multiple-quantum correlations; TOCSY, total correlation spectroscopy; s, singlet; d, doublet; dd, double of doublet; t, triplet; m, multiplet

1. Tsugawa H, Cajka T, Kind T, Ma Y, Higgins B, Ikeda K et al. MS-DIAL: data-independent MS/MS deconvolution for comprehensive metabolome analysis. *Nat Methods* **2015**, 12(6), 523-526.
